# Therapeutic Potential of 7,8-Dimethoxycoumarin in Tumor Necrosis Factor-Alpha-Induced Trigeminal Neuralgia in a Rat Model

**DOI:** 10.3390/cimb47070518

**Published:** 2025-07-04

**Authors:** Nallupillai Paramakrishnan, Kanthiraj Raadhika, Sumitha Elayaperumal, Yuvaraj Sivamani, Yamunna Paramaswaran, Lim Joe Siang, Thiagharajan Venkata Rathina Kumar, Khian Giap Lim, Muthusamy Ramesh, Arunachalam Muthuraman

**Affiliations:** 1 Department of Pharmacognosy, JSS College of Pharmacy, Mysuru, JSS Academy of Higher Education and Research, Mysuru 570015, Karnataka, India; 2 Institute of Pharmacology, Madurai Medical College, Madurai 625020, Tamil Nadu, India; 3 Department of Biotechnology and Bioinformatics, JSS Academy of Higher Education and Research, Mysuru 570015, Karnataka, India; 4 Crescent School of Pharmacy, B.S. Abdur Rahman Crescent Institute of Science and Technology, Chennai 600048, Tamil Nadu, India; yuvaraj@crescent.education; 5 Faculty of Pharmacy, AIMST University, Jalan Bedong-Semeling, Bedong 08100, Kedah, Malaysia; pyamunna97@gmail.com (Y.P.);; 6 Department of Pharmacognosy, College of Pharmacy, Madurai Medical College, Madurai 625020, Tamil Nadu, India; 7 Clinical Pharmacy & Pharmacy Practice Unit, Faculty of Pharmacy, AIMST University, Jalan Bedong-Semeling, Bedong 08100, Kedah Darul Aman, Malaysia; 8 Department of Pharmaceutical Analysis, Omega College of Pharmacy, Hyderabad 501301, Telangana, India; 9 Department of Pharmacology, Akal Toxicology Research Centre, Akal College of Pharmacy & Technical Education, Mastuana Sahib, Sangrur 148001, Punjab, India; 10 Pharmacology, Toxicology and Basic Health Sciences Unit, Faculty of Pharmacy, AIMST University, Jalan Bedong-Semeling, Bedong 08100, Kedah, Malaysia

**Keywords:** allodynia, endoneural injection, hematoxylin staining, hyperalgesia, reduced glutathione

## Abstract

Trigeminal neuralgia is a chronic pain disorder due to neuronal damage. The present study was designed to investigate the effect of 7,8-dimethoxy coumarin (DMC) in a rat model of trigeminal neuralgia. The neuropathic pain was induced by the single endoneural injection of tumor necrosis factor-alpha (TNF-α; 0.1 μL: stock 10 pg/mL) in the rat trigeminal nerve. The DMC (100 and 200 mg/kg) and carbamazepine (100 mg/kg) were administered orally for 10 consecutive days from the 5th day of TNF-α injection. The battery of behavioral tests, i.e., acetone drop and Von Frey filament test, was performed to assess the degree of thermal and mechanical allodynia on 0, 1st, 7th, and 14th days. In addition, the biochemical tests, i.e., total protein, thiobarbituric acid reactive substances (TBARS), reduced glutathione (GSH), and TNF-α, were also performed in trigeminal nerve tissue. Furthermore, TNF-α-induced neuronal histopathological changes were also evaluated by the eosin and hematoxylin staining method. The administration of DMC was shown to demonstrate the significant (*p* < 0.05) reversal of TNF-α-induced percentage reduction of thermal and mechanical sensitivity, along with a rise in TBARS and TNF-α and a decrease in GSH levels. Further, DMC also attenuates the histopathological changes. It may be concluded that DMC may be a potential therapeutic agent for the management of trigeminal neuralgia disorders.

## 1. Introduction

According to the International Association for the Study, pain is defined as “an unpleasant sensory and emotional experience associated with, or resembling that associated with, actual or potential tissue damage” [1]. Based on the duration, pain is classified as acute or chronic pain. Further, it is also categorized as nociceptive, nociplastic pain, and neuropathic pain, with causes and characteristic features of neuronal pathogenesis [2]. Nociceptive pain is known to be pain raised in the damaged part of the tissue, a sensory neuron with noxious stimuli associated with pain receptor activation. Nociplastic pain is known to be pain raised in the altered nociception of sensory neurons without damage to the tissue [3]. Neuropathic pain is known to be pain raised in the peripheral or central nervous system due to the primary lesion, nerve dysfunction, or transitory perturbation of neuronal tissue [4]. The neuropathic pain symptoms are two main categories, i.e., positive symptoms like allodynia, paresthesia, dysesthesia, and hyperalgesia; and negative symptoms like hypoesthesia and hypoalgesia [5,6]. Sometimes patients also express the feeling of burning, numbness, a feeling of needle-sharp stabbing, throbbing pain, tingling, and weakness [7].

Trigeminal neuralgia affects the trigeminal nerve, and it causes disabilities and incapacities with intense, recurring pain episodes. The primary causes of trigeminal neuralgia are neurovascular compression, which leads to presses the nerve-blood vessel on the nerve root [8]. Furthermore, the following factors also contribute to the disabilities and incapacities with trigeminal neuralgia, i.e., severe, sudden, and often debilitating facial pain; impact on daily activities, i.e., difficulty to speak, eat, drink, brush teeth, shave, and difficulty maintaining personal hygiene; social isolation; impact mental health, i.e., anxiety, depression, cognitive dysfunction, and suicidal thoughts; difficulty to maintain employment; and financial hardship [9,10]. According to the National Institutes of Health (NIH), the global prevalence rate of trigeminal neuralgia is between 0.16% and 0.3% of the total population. This estimated rate of trigeminal neuralgia is rising between 0.03% to 0.3% yearly. The higher prevalence rate of trigeminal neuralgia is raised among women older than 40 years [11].

Neuropathic pain is mainly managed with the treatment of sodium channel blockers, i.e., carbamazepine (CBZ), lamotrigine, oxcarbazepine, valproate; selective serotonin reuptake inhibitors, i.e., citalopram and paroxetine; dual inhibition of norepinephrine and dopamine reuptake, i.e., bupropion; N-methyl-d-aspartate (NMDA) type of glutamate receptor antagonists, i.e., ketamine, memantine, and dextromethorphan; N-type calcium channel blockers, i.e., pregabalin and gabapentin, and mirogabalin; inhibitor peripheral neuronal sodium channel blocker, i.e., lidocaine; opioid receptor agonist, i.e., tramadol; and topical lidocaine and activator of transient receptor potential cation channel vanilloid—1 (TRPV1) receptor, i.e., topical capsaicin [12,13,14].

CBZ is widely used for the facial and trigeminal neuralgia [15]. These agents regulate the neuronal membrane hyper-excitability [16]; ectopic discharges and neuronal sensitization [17]; denervation supersensitivity [18]; and loss of inhibitory controls [19]. However, it partially relieves the pain symptoms. Furthermore, there are no single treatment agents that are safe and have better efficacy for the treatment of various neuropathic pain conditions [20,21].

TNF-α is one of the pro-inflammatory cytokines, and it causes the development of neurodegeneration and neuropathic pain. It also causes neuronal excitation and synaptic plasticity via the elevation of glutamate in neuronal-glial cells [22]. However, the treatment of TNF-α synthesis inhibitors, i.e., 3,6′-dithiothalidomide; TNF-α receptor blockers like etanercept; and inhibitors of TNF-α activity, i.e., adalimumab, certolizumab, infliximab, and golimumab, demonstrates the neuroprotection [23,24,25]. Furthermore, CBZ also possesses the reduction of neuronal TNF-α expression [26]. Moreover, the inhibitors of TNF-α increase the risk of infections, and serious complications to cardiac and neuronal tissues [24]. Plant-based medicines are promising the inhibition of TNF-α and neuroprotection [27]. Further, the natural neuroprotective agent is needed for the inhibition of TNF-α synthesis, TNF-α activity in tissue (cells), and receptor binding actions [28,29].

Phytomedicines like resveratrol, curcumin, sesamol, shogaol, paradol, and equol possess TNF-α inhibitory actions that lead to the production of neuroprotective effects [30,31,32,33]. 7,8-dimethoxycoumarin (DMC) is a coumarin derivative obtained from *Artemisia caruifolia*. Furthermore, it possesses the potential anti-oxidant and anti-inflammatory action [34]. Another coumarin derivative, i.e., 7,8-dihydroxycoumarin, demonstrates neuroprotection against sciatic nerve injury [35]. Furthermore, numerous studies suggested that 6,7,8-trimethoxycoumarin and 5,7-dimethoxycoumarin reduce the vincristine induced neuropathic pain via reduction of the elevated levels of plasma and tissue TNF-α content [36,37]. Furthermore, the therapeutic potential of DMC was proven against the various toxin-associated tissue injuries [38]. However, the natural medicine, i.e., DMC, effect on neuroprotection against the TNF-α-mediated nerve injury has not been explored yet. Based on this literature evidence, DMC was selected for the exploration of anti-neuralgic action of DMC against the TNF-α-induced neuropathic pain. DMC possesses several advantages over the coumarin compounds due to its potential anti-inflammatory and antioxidant properties. Furthermore, it is better tolerated and exhibits a wider range of therapeutic benefits [39]. Hence, the present study is designed to investigate the therapeutic potential of DMC in TNF-α-induced trigeminal neuralgia in rats.

## 2. Materials and Methods

### 2.1. Animals Used

The disease-free male Wistar rats [middle-aged animals; 12–14 months] with 200–230 g of body weight were used. Animals were fed a standard laboratory diet and allowed access to drinking water ad libitum. The animals were maintained in the central animal house with a 12 h day and night cycle. The institutional animal ethics committee (IAEC approval no.: 1407/a/11/CPCSEA; Duration of 04.05.2013 to 3 November 2013, Akal Toxicology Research Centre, Akal College of Pharmacy & Technical Education, Mastuana Sahib, Sangrur-148001, Punjab, India) authorized this experimental design. The experiments were conducted according to the IAEC guidelines and adhered to international regulations regarding the use of animals in experimentation. Furthermore, the 3Rs principle was adopted for this animal experimentation.

### 2.2. Induction of Trigeminal Neuralgia

**Figure 1:**
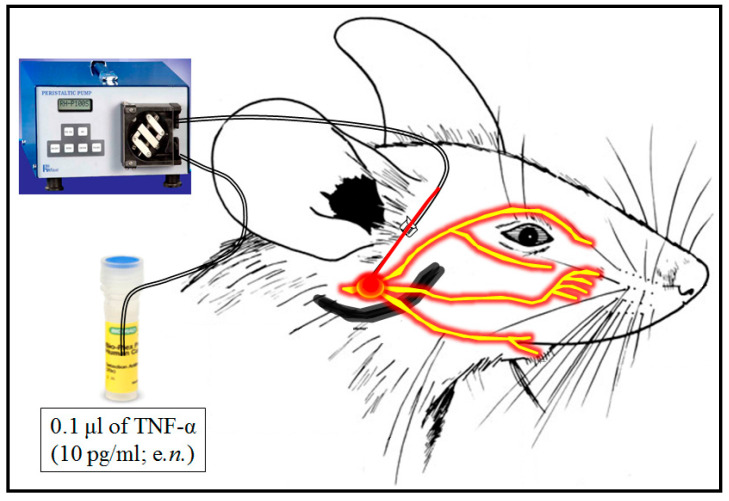
Endoneurial injection of TNF-α in rat trigeminal neurons.

Induction of trigeminal neuralgia was induced by endoneurial injection of TNF-α as described method of Wagner and Myers [40] with a slight modification of Sorkin and Doom [41]. Furthermore, TNF-α expression and accumulation are known to modulate the neuronal calcium channels, which leads to neuronal excitation and neuropathic pain [42]. Hence, the endoneurial injection of TNF-α method was adopted in this study for the induction of trigeminal neuralgia. Briefly, the rats were anesthetized by intraperitoneal injection of thiopentone sodium (35 mg/kg; i.p.). The hair of the right mandibular bone region was removed, a topical antiseptic solution (Betadine Antiseptic Liquid) was applied at once, and the surface was cleaned with sterile cotton. The trigeminal nerve was exposed by the opening of the facial surface skin and superficial fascia, i.e., between the lower segment line (i.e., 5 mm) of the right eye and right ear of the rat [43]. Then, a 35-gauge needle was inserted into the endoneural space of the right trigeminal nerve, and the other end of the needle was connected to the Ravel peristaltic pump (Ravel Hiteks Pvt. Ltd., Chennai, India). The single endoneural injection of TNF-α (i.e., 0.1 μL from 10 pg of TNF-α/mL with a speed of 0.1 μL/min; i.e., 1 pg) was injected as described by Damjanovska et al. [44]. Thereafter, the needle was removed, and the skin incision was closed by using the non-absorbable silk thread (4–0). The sham group of animals underwent all surgical procedures, i.e., skin incision, needle application, and closure of the skin surface, except endoneurial injection of TNF-α. The endoneurial injection of TNF-α in the trigeminal nerve region was illustrated in Figure 1.

### 2.3. Experimental Design

The experimental design consisted of seven groups. Each group consists of 8 Wistar rats. With consideration to ensure the scientific and ethical validity of this study, the six animals were used for the biochemical estimations, and the remaining two animals were used for the histopathology. The same tissue cannot be used for both the biochemical and histopathology assessments. Hence, 8 rats were used in each group. Group I: Animals were used as naive controls, which did not receive any drug or TNF-α administration. Group II: Animals were served as a sham group. This group underwent all surgical procedures except endoneurial injection of TNF-α. It helps to discriminate the neuropathic pain changes due to the endoneurial injection of TNF-α (group III), not due to the surgical procedure. Group III: Animals were employed as a negative group. This group of animals was employed for the endoneural injection of TNF-α (0.1 μL of 1 pg of TNF-α/min) in the rat trigeminal nerve as described in the previous section. Group IV: Animals were employed as vehicle groups. This group of animals was administered orally (p.o.) 2.5 mL/kg of 0.5% *w*/*v* of carboxymethyl cellulose (CMC) solution for 10 consecutive days in TNF-α-treated animals. Experimentally, CMC does not show any pharmacological effect. Hence, DMC and carbamazepine, for this study, were prepared by using CMC as the medium of drug preparation. It helps to discriminate the effect of drugs in neuropathic pain disorders and the vehicle group effects. Group V and VI: Animals were employed as test drug-treated groups. This group of animals was administered DMC (100 and 200 mg/kg; p.o., respectively) for 10 consecutive days in TNF-α-treated animals. DMC was obtained from MedChemExpress (Allianz BioInnovation, Mumbai, India). The bioavailability of DMC is not explicitly stated in the experimental research data. However, it reported to show the therapeutic potential effects with a variable dose range, i.e., 50, 75, and 100 mg/kg [38,45]. Based on this literature report, the doses of DMC were selected for this study.

**Figure 2:**
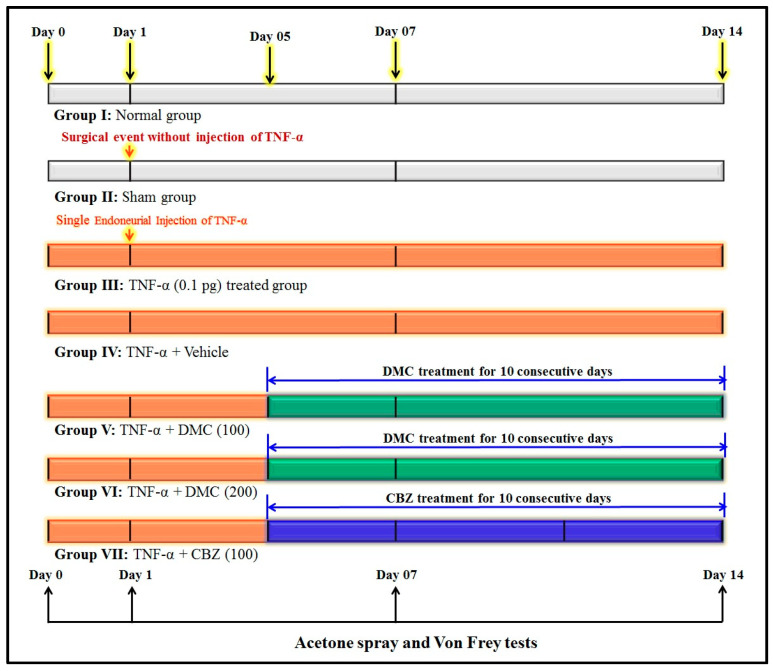
Experimental design for TNF-α-induced trigeminal neuralgia in rats.

Group VII: Animals were employed as reference drug-treated groups. This group of animals was administered carbamazepine (CBZ; 100 mg/kg; p.o.) for 10 consecutive days in TNF-α-treated animals. The changes of TNF-α and drug therapy-associated pain behavioral changes in the acetone spray test and Von Frey test were assessed at different time intervals, i.e., 0, 1, 7, and 14th days. On the 14th day, animals were sacrificed by the cervical dislocation method. Furthermore, the trigeminal nerve tissue biomarker changes, i.e., thiobarbituric acid reactive substances (TBARS), reduced glutathione (GSH), TNF-α, and total proteins were estimated. Furthermore, TNF-α-associated trigeminal nerve histopathological changes were also evaluated by the eosin and hematoxylin staining method. The flow chart of the experimental design is illustrated in Figure 2.

### 2.4. Behavioural Assessment

The TNF-α-induced pain behavior changes in rats were assessed by the acetone drop test and Von Frey hair filament test on 0, 1st, 7th, and 14th days. The specially designed rat restraint holder was arranged for the assessment of pain behaviors in rats. This rat restraint holder covers the trunk portion of the rat body and freely allows 360° head movement outside of the chamber. It helps to apply the acetone drops and Von Frey hair filaments in the region of the whisker pad (vibrissae) during the pain assessment. The details of the acetone spray test and Von Frey test were described in the following sections.

#### 2.4.1. Acetone Drop Test

The acetone drop test was used for the assessment of chemical cold allodynia type of pain sensation in peripheral nerve endings, as described method of Yoon et al. [45], with a slight modification from Gupta et al. [46]. Briefly, 40 µL of acetone solution was applied to the orofacial whisker pad regions using a single-channel 100 µL micropipette (Benchmark Scientific Inc., Sayreville, NJ, USA). After applying the acetone solution, the animals’ responses to the acetone solution were noted with a standard score, i.e., score 0: no response; score 1: head shaking; score 2: head shaking and rubbing; score 3: rubbing with time intervals; and score 4: continuous rubbing. The maximal cumulative cold chemical sensitivity score (1 + 2 + 3 + 4 = 10) was noted as 10. Based on this cumulative score, the percentage reduction of thermal sensitivity was assessed. The increased cold chemical sensitivity score and its percentage value indicate that TNF-α causes trigeminal neuralgia, whereas a reduction of percentage cold chemical sensitivity was noted as an improvement of trigeminal neuralgia.

#### 2.4.2. Von Frey Hair Filament Test

The Von Frey hair filament test was used for the assessment of mechanical allodynia type of pain sensation in peripheral nerve endings as described method of Chaplan et al. [47], with a slight modification of Gupta et al. [46]. Briefly, the animals were placed in a rat restraint holder, and Von Frey hair filament (North Coast Medical & Rehabilitation products, Morgan Hill, CA, USA) was applied to the orofacial whisker pad regions with a variable bending force (pre-determined) filaments, i.e., 5.9–98 mN (≅ 0.59–9.8 g) for rats. The filaments were selected from lowest to highest and applied tactile movement with the bending of filaments 10 times in orofacial whisker pad regions, with a 10 s time frequency. There was no response to 10 times of stimuli; the specific filament response was considered normal. If 10 responses were received, the specific filament force was considered as the peak level of pain response. A minimum of two responses were required from the selected filaments for the assessment of the % mechanical sensitivity response [48]. The % of mechanical sensitivity response (% MSR) was assessed as per the following equation:

% MSR = 100 × ( PWT − 1)/(10 − 1)

Here, MSR, mechanical sensitivity response; PWT, paw withdrawal threshold; and an arbitrary value of 10 (an arbitrary value of the maximum possible value).

### 2.5. Biochemical Estimation

On the 14th day, animals were sacrificed by the cervical dislocation method. The trigeminal nerve tissue was isolated and homogenized with an ice-cold phosphate buffer (pH 7.4) solution. The clear supernatant was collected by centrifugation at a 769 G force. This aliquot was used for the assessment of tissue biomarkers, i.e., thiobarbituric acid reactive substances (TBARS), reduced glutathione (GSH), TNF-α, and total protein levels.

#### 2.5.1. Estimation of TBARS Level

The TBARS was estimated as the method described by Niehaus and Samuelsson [48]. The principle of this method was to represent the formation of free radical-associated lipid peroxidation products, i.e., malondialdehyde (MDA). Briefly, the 1 mL of aliquot was mixed with 2.0 mL of trichloroacetic acid (TCA)—thiobarbituric acid (TBA)—hydrochloric acid (HCL) reagent mixture (TTH) in a 15 mL test tube. The TTH reagent mixture was prepared by mixing of 1:1:1 ratio of 5% TCA; 0.25 N of HCl; and 0.375% TBA. The test tubes were placed in a boiling water bath for 15 min. Then, the tubes were cooled under tap water. If any precipitation was found in the test tubes, the tubes were centrifuged at 1000× *g* for 10 min. The changes of pink-colored chromogen were measured by a spectrophotometer (DU 640B Spectrophotometer, Beckman Coulter Inc., Brea, CA, USA) at a 535 nanometer wavelength. The reference standard plot was prepared with 0 to 2.5 nmol of 1,1′,3,3′-tetramethoxy propane per milliliter. The result was reported as nmol of TBARS per nmol per milligram of protein.

#### 2.5.2. Estimation of GSH Level

The GSH was estimated as the method described by Beutler et al. [49]. The principle of this method represents the formation of free radicals and the neutralizing capacity of endogenous free radical scavenging molecules, i.e., reduced glutathione. Briefly, the 0.5 mL of the aliquot was mixed with 2 mL of 0.3 M disodium hydrogen phosphate. Thereafter, 0.25 mL of 0.001 M of freshly prepared 5,5′-dithiobis (2-nitrobenzoic acid) (DTNB). Then, test tubes were vortexed gently for 2 min. The changes of yellow-colored chromogen were measured by a spectrophotometer (DU 640B Spectrophotometer, Beckman Coulter Inc., Brea, CA, USA) at a 412 nanometer wavelength. The reference standard plot was prepared with 10 to 100 μg of reduced glutathione per milliliter. The result was reported as μmol of GSH per μg per milligram of protein.

#### 2.5.3. Estimation of TNF-α Level

The TNF-α was estimated as a method described by Muthuraman and Ramesh [50] by the enzyme-linked immunosorbent assay (ELISA) method. The TNF-α test procedure was followed as per the rat TNF-α ELISA kit (E-EL-R2856; Elabscience Bionovation Inc., New Delhi, India) instructions. Briefly, about 100 μL tissue aliquot was placed in a TNF-α antibody pre-coated microplate well and incubated for 90 min at room temperature (37 °C), then washed 3 times with 100 μL of washing buffer. Thereafter, 100 μL of streptavidin-horseradish peroxidase (HRP) enzyme solution was added and incubated for 30 min at room temperature, and then washed 5 times. Furthermore, 90 μL of substrate solution was added, and the incubation was performed for 15 min at room temperature. Crucially, 50 μL of stop solution was added to stop the TNF-α reaction. The changes of yellow chromogen were measured by a microplate reader (Bio-Tek Microplate Instruments, Butterworth, Penang, Malaysia) at a 450 nanometer wavelength. The reference standard plot was prepared with standard 15.63–1000 pg of TNF-α per milliliter. The result was reported as a pg of TNF-α per mg of tissue protein.

#### 2.5.4. Estimation of Tissue Total Proteins

The tissue total proteins were estimated as described by Lowry et al. [51]. In short, about 0.15 mL of the aliquot was mixed with 1 mL of phosphate buffer and 5 mL of Lowry’s reagents in the test tubes. The tubes were incubated for 15 min at room temperature. Thereafter, 0.5 mL of Folin–Ciocalteu reagent was added and vortexed rapidly. The incubation again continued for 30 min at room temperature. The changes of purple-colored chromogen were measured by a spectrophotometer (DU 640B Spectrophotometer, Beckman Coulter Inc., Brea, CA, USA) at 750 nanometer wavelength. The reference standard plot was prepared with 0.2–2.4 mg of bovine serum albumin per milliliter. The result was reported as mg of tissue total proteins per gram of tissue.

#### 2.5.5. Evaluation of Histopathological Changes

The TNF-α-induced trigeminal nerve histopathological changes in rats were assessed by eosin-hematoxylin techniques as described method of Rao et al. [52] with a slight modification of Borin et al. [53]. Briefly, tissue was fixed in 10% formalin solution and cut into transverse sections at 4 μm thickness. We ensured the trigeminal nerve portion was fixed for the preparation of the transverse section of the tissue. The tissue histopathological changes were observed, and images were captured by using an Olympus microscopic camera EP50 (Olympus Corporation, Tokyo, Japan). Microscopic examinations were performed under a 400× light microscope, scale bar 35 µm.

### 2.6. Statistical Analysis

All the data were presented as the standard deviations (SD: n = 6). The acetone drop test and Von Frey hair filament test data were statistically analyzed by a two-way analysis of variance (ANOVA) test, accompanied by the Bonferroni post hoc test using Graphpad Prism software version 5.0 Dotmatics (R&D Scientific Software Company, San Diego, CA, USA). Furthermore, the data of TBARS, GSH, TNF-α, and total protein levels were analyzed by one-way ANOVA followed by Tukey’s multiple range tests using Graphpad Prism software version 5.0 Dotmatics (R&D Scientific Software Company, San Diego, CA, USA). The probability (*p*) value less than 0.05 was considered statistically significant.

## 3. Results

### 3.1. Effect of DMC in TNF-α-Induced Acetone Drop Test

**Figure 3:**
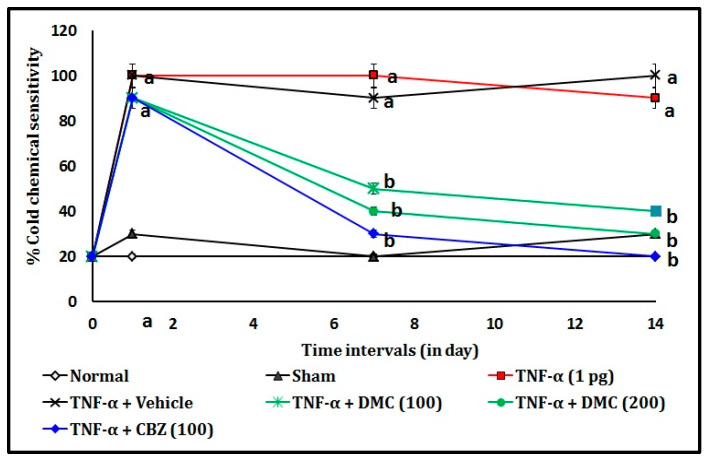
Effect of DMC in TNF-α-induced acetone drop test. Digits in parentheses indicate the dose in mg/kg. Data expressed as mean ± SD, n = 6 rats per group. ^a^ *p* < 0.041 vs. sham group. ^b^ *p* < 0.037 vs. TNF-α-treated group. Abbreviations: CBZ stands for carbamazepine; DMC stands for 7,8-dimethoxy coumarin; pg, picogram; and TNF-α stands for tumor necrosis factor-alpha.

The endoneural injection of TNF-α (1 picogram; pg) in rats showed a significant (*p* < 0.05) rise in percentage cold chemical sensitivity when compared to the normal control group. It indicates that TNF-α causes the potential trigeminal neuralgia associated with thermal (chemical) sensation in trigeminal nerve endings. The oral administration of DMC (100 and 200 mg/kg; p.o. for 10 consecutive days) attenuates the TNF-α-induced trigeminal neuralgia in a dose and time-dependent manner when compared to the TNF-α-administered group. This effect resembled that of the CBZ (100 mg/kg; p.o. for 10 consecutive days) in TNF-α-treated animals. The results of the DMC in TNF-α-induced cold chemical sensitivity changes are illustrated in Figure 3.

### 3.2. Effect of DMC in TNF-α-Induced Von Frey Hair Test

**Figure 4:**
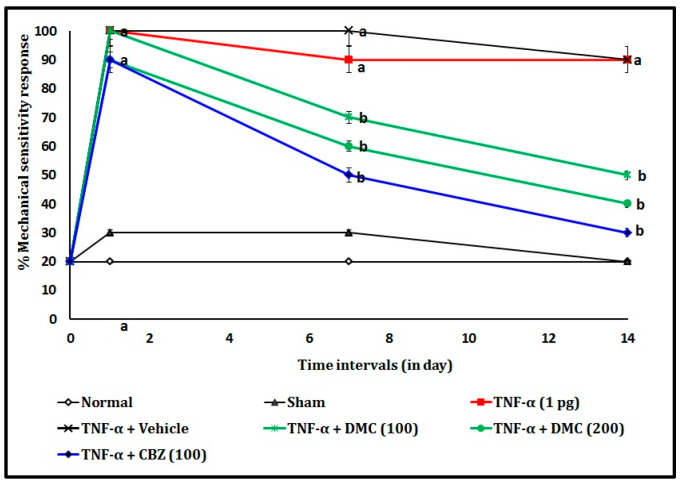
Effect of DMC in TNF-α-induced Von Frey hair test. Digits in parentheses indicate the dose in mg/kg. Data expressed as mean ± SD, n = 6 rats per group. ^a^ *p* < 0.043 vs. sham group. ^b^ *p* < 0.041 vs. TNF-α-treated group. Abbreviations: CBZ stands for carbamazepine; DMC stands for 7,8-dimethoxy coumarin; pg, picogram; and TNF-α stands for tumor necrosis factor-alpha.

The endoneural injection of TNF-α (1 pg) in rats showed a significant (*p* < 0.05) increase of % mechanical sensitivity response when compared to the normal group. It indicates that TNF-α causes the potential trigeminal neuralgia associated with mechanical sensation in trigeminal nerve endings. The oral administration of DMC (100 and 200 mg/kg; p.o. for 10 consecutive days) attenuates the TNF-α-induced trigeminal neuralgia in a dose and time-dependent manner when compared to the TNF-α-administered group. This effect resembled that of the CBZ (100 mg/kg; p.o. for 10 consecutive days) in TNF-α-treated animals. The results of the DMC in TNF-α-induced mechanical sensitivity changes are illustrated in Figure 4.

### 3.3. Effect of DMC in TNF-α-Induced Tissue Biomarker Changes

The endoneural injection of TNF-α (1 pg) in rats showed a significant (*p* < 0.05) rise in the TBARS and TNF-α levels and decreased the GSH level when compared to the normal group. It indicates that TNF-α causes the potential trigeminal neuralgia associated with the biomarker changes in the trigeminal nerve. The oral administration of DMC (100 and 200 mg/kg; p.o. for 10 consecutive days) attenuates the TNF-α-induced tissue biomarker changes dose-dependent manner when compared to the TNF-α-administered group. DMC (100 and 200 mg/kg) showed the reduction of TBARS and TNF-α levels and reversed the proposed GSH levels against the TNF-α toxicity. The effective antioxidant treatment of DMC has been shown to restore GSH levels, and it helps to counteract the TNF-α-induced oxidative stress. This effect resembled that of the CBZ (100 mg/kg; p.o. for 10 consecutive days) in TNF-α-treated animals. The results of the DMC in TNF-α-induced biochemical changes are mentioned in Table 1.

### 3.4. Effect of DMC in TNF-α-Induced Histopathological Changes

**Figure 5:**
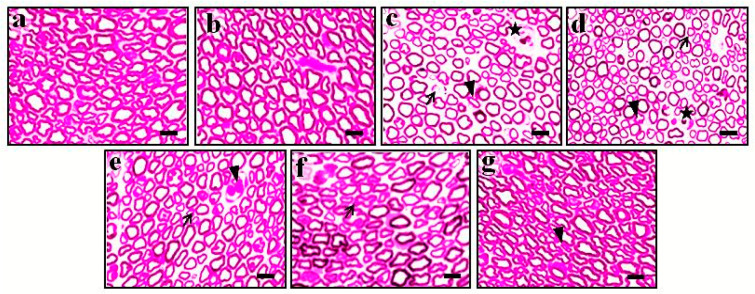
Effect of DMC in TNF-α-induced histopathological changes of trigeminal nerve tissue in rats. In each group, two rats were used for the assessment of neuronal histopathological changes. Tissue sections were stained with the eosin and hematoxylin staining methods. (**a**–**g**) shows histological changes of neuronal tissue of normal, sham, TNF-α (1 pg; endoneural injection), vehicle, DMC (100 mg/kg; for 10 days), DMC (200 mg/kg; for 10 days), and CBZ (100 mg/kg; p.o., for 10 days) administered groups, respectively. (**a**,**b**) show the normal structure of neuronal tissue. (**c**) shows the TNF-α-induced axonal degeneration (thin arrow), neuronal hypertrophy (star), and neurovascular injury (arrowhead). (**d**,**g**) show that the DMC and CBZ possess the potential neuroprotective actions against TNF-α toxicity. Microscopic examinations were performed under a 400× magnification, scale bar 35 µm.

The histological changes of trigeminal nerve tissue in the normal group showed no changes, whereas the endoneural injection of TNF-α (1 pg) was shown to have potential microscopical changes in trigeminal nerve, i.e., axonal degeneration, neuronal hypertrophy, and neurovascular injury. However, the oral administration of DMC (100 and 200 mg/kg; p.o. for 10 consecutive days) showed the potential amelioration of TNF-α-induced histopathological changes in neuronal tissue. The results were similar to the reference drug, i.e., CBZ (100 mg/kg; p.o. for 10 consecutive days) treatment groups. It indicates that DMC possesses the potential neuroprotective action against TNF-α-induced neuronal damage and its dysfunctions. The changes were observed under 400× magnification (scale bar: 35 µm). The effects of DMC in TNF-α-induced histopathological changes are depicted in Figure 5.

## 4. Discussion

The endoneural injection of TNF-α (1 pg) in rats demonstrated a significant (*p* < 0.05) increase in percentage cold chemical sensitivity and % mechanical sensitivity response, along with raising the TBARS and TNF-α levels; and decreasing the GSH level. Furthermore, it also alters the trigeminal nerve histological features. It indicates that TNF-α causes the potential trigeminal neuralgia with trigeminal nerve inflammatory neurodegenerations. The oral administration of DMC (100 and 200 mg/kg; p.o. for 10 consecutive days) attenuates the TNF-α-induced above pain behavior, biomarkers, and histological changes in a dose-dependent manner with a similar effect to the treated group. This indicates that DMC has potential neuroprotective effects with multiple cellular mechanisms.

The general mechanism of neuropathic pain pathogenesis was altered by the neuronal transmission of nerve impulses via ion channel dysregulation, especially voltage-gated cation channels, leading to lower poly-synaptic nerve response and inhibiting the post-synaptic potentiation [54,55]. The nerve injury readily releases inflammatory cytokines like TNF-α, which leads to further acceleration of inflammatory reactions and enhances cellular oxidative stress [56,57]. Subsequently, oxidative stress and free radicals are known to cause membrane lipid peroxidation and alter the neuronal membrane potentials. In this condition, the cellular endogenous antioxidant system fails to handle the oxidative stress, causing neurodegeneration and loss of neuronal plasticity functions [58]. A similar pathogenesis was involved in the TNF-α-induced neuropathic pain conditions in experimental animal models [59]. Our present data also proved that endoneural injection of TNF-α causes potential trigeminal neuralgia. In the acute phase, TBARS is a primary marker for the membrane lipid peroxidation process, which occurs when the cytosolic free radical generation and calcium accumulation [60]. Normally, free radicals are scavenged by GSH molecules, whereas the abundant generation of free radicals from mitochondria during abnormal neuron cell metabolism, the scavenging mechanism due to a lack of GSH molecules [61]. Furthermore, this situation accelerates the expression of neuronal prion proteins, like apoptotic proteins and neuronal cytoskeletal proteins, which leads to neuronal damage and neurodegeneration [62,63]. In the present study, biomarkers and histological assessment revealed the same results in TNF-α-induced trigeminal neuralgia in rats.

The coumarin derivative, i.e., DMC, possesses free radical scavenging and the prevention of inflammatory reactions [34]. Our previous study also revealed that DMC possesses anti-secretory and anti-inflammatory action [45], and antioxidant and regulation of mitochondrial permeability transition pore opening [38]. Furthermore, another coumarin derivative, i.e., 7,8-dihydroxycoumarin reported to demonstrate the neuroprotective action against sciatic nerve injury in mice. It also promotes the repair process of injured nerves via the upregulation of growth-associated protein 43 expression [35]. Moreover, 5,7-dimethoxy coumarin (citropten) also evidenced to attenuate the vincristine-induced neuropathic pain in male BALB/c mice by reversing elevated hippocampal serotonin, inosine, and dopamine; and striatal serotonin levels, 5HT3 receptors 5-HT3 receptor antagonistic action [37]. Further, citropten showed antidepressant neuroprotection against chronic mild stress-induced depression in rats via raising the heat shock protein-70 expression and monoamine oxidase-A inhibitory actions [64]. In vitro study revealed that DMC ameliorates the TNF-α-induced damage of human keratinocyte HaCaT cells via inhibition of Nuclear factor kappa B activation and phosphorylation of mitogen-activated protein kinase-like c-Jun N-terminal kinases and extracellular-signal-regulated kinase [39]. Current literature also revealed that 7-methoxy coumarin possesses the potential for neuroprotection and amelioration of neuropathic pain via inhibition of neuronal phospholipase enzyme and inhibition of the neuronal voltage-gated calcium channels [65].

**Figure 6:**
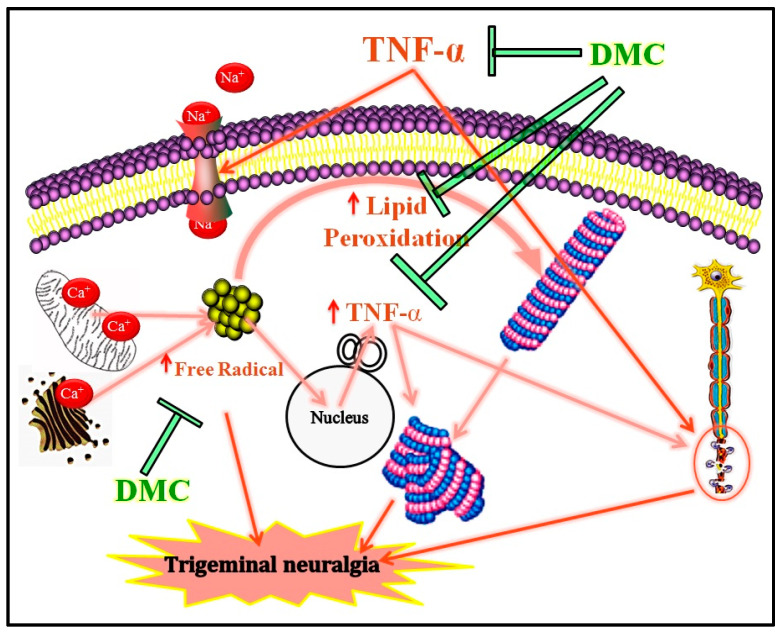
Salient findings of DMC in TNF-α-induced trigeminal neuralgia. TNF-α administration alters the neuronal sodium channel, leads to the release the stored calcium ions from mitochondria and endoplasmic reticulum, which leads to an enhancement the cellular free radicals, expression of TNF-α, and alters the neuronal cytoskeletal protein, i.e., tubular proteins (purple color spindle structure). Crucially, it causes neuronal damage and neurodegeneration. Whereas, the DMC administration shows the inhibition of TNF-α-induced generation of free radicals, lipid peroxidation, and neurodegeneration. Abbreviations: DMC stands for 7,8-dimethoxy coumarin, and TNF-α stands for tumor necrosis factor-alpha.

DMC is known to reduce the expression of TNF-α levels against ischemic reperfusion injury [38]. Furthermore, coumarin derivatives also reduce the expression of inflammatory cytokine, i.e., TNF-α, leading to the anti-inflammatory reactions in vitro and in vivo [66]. The primary mechanism of the reduction of TNF-α levels is mainly due to the inactivation of the nuclear transcription factor kappa-B (NF-κB) and mitogen-activated protein kinase (MAPK) expression pathways [67]. Our study revealed that DMC potentially inhibits the TNF-α level in the trigeminal nerve and TNF-α-induced neuropathic pain behavior in the rat. The major limitations are that this study showed that DMC therapeutic effects were explored in trigeminal neuralgia in rat models with a minimal tissue histology sample size. Hence, DMC effects must be extended to higher vertebrate animals with large populations before entering into the human trials. The salient finding of DMC in TNF-α-induced trigeminal neuralgia is illustrated in Figure 6.

## 5. Conclusions

DMC has potential therapeutic action for the amelioration of trigeminal neuralgia via inhibition of free radical scavenging, reduction of lipid peroxidation, and enhancement of the endogenous antioxidant molecule actions. Hence, DMC may have potential for use for the trigeminal neuralgia due to its potential antioxidant, anti-inflammatory, and anti-TNF-α actions. Hence, DMC may be a good candidate for further studies in the various animal models and perhaps in humans in the future.

## Figures and Tables

**Table 1:** Effect of DMC in TNF-α-induced tissue biomarker changes.

Groups	TBARS (nmol/mg of Protein)	GSH (µg/mg of Protein)	TNF-α (pg/mg of Protein)
Normal	3.72 ± 0.84	73.64 ± 2.92	5.04 ± 0.02
Sham	4.08 ± 0.59	70.93 ± 1.86	4.98 ± 0.09
TNF-α (0.1 pg)	19.48 ± 0.26 ^a^	43.17 ± 2.46 ^a^	28.84 ± 0.07 ^a^
TNF-α + Vehicle	18.71 ± 0.59	45.64 ± 2.34	28.61 ± 0.04
TNF-α + DMC (100)	10.02 ± 0.35 ^b^	61.92 ± 1.69 ^b^	11.48 ± 0.08 ^b^
TNF-α + DMC (200)	8.11 ± 0.37 ^b^	64.73 ± 1.83 ^b^	9.72 ± 0.07 ^b^
TNF-α + CBZ (100)	5.73 ± 0.28 ^b^	68.18 ± 2.47 ^b^	6.35 ± 0.03 ^b^

Digits in parentheses indicate the dose in mg/kg. Data expressed as mean ± SD, n = 6 rats per group. ^a^ *p* < 0.05 vs. sham group. ^b^ *p* < 0.05 vs. TNF-α-treated group. Abbreviations: CBZ stands for carbamazepine; DMC stands for 7,8-dimethoxy coumarin; pg, picogram; GSH stands for reduced glutathione; TBARS stands for thiobarbituric acid reactive substances; and TNF-α stands for tumor necrosis factor-alpha.

## Data Availability

The data presented in this study are available on request from the corresponding author.

## Abbreviations

The following abbreviations are used in this manuscript:

DMC	7,8-dimethoxy coumarin
TNF-α	Tumor necrosis factor-alpha
TBARS	Thiobarbituric acid reactive substances
GSH	Reduced glutathione
CBZ	Carbamazepine
NMDA	N-methyl-d-aspartate
IAEC	Institutional animal ethics committee
p.o.	Per oral
TCA	Trichloroacetic acid
TBA	Thiobarbituric acid
HCL	Hydrochloric acid
DTNB	5,5′-dithiobis (2-nitrobenzoic acid)
SD	Standard deviations
ANOVA	Analysis of variance
pg	picogram

## References

[1] Breton-Piette A., Gélinas C., Aita M. (2025). The complexity of the neonatal pain construct: A commentary on Glenzel et al. (2023). Pain Manag. Nurs..

[2] Macionis V. (2025). Nociplastic Pain: Controversy of the Concept. Korean J. Pain.

[3] Johnston K.J.A., Signer R., Huckins L.M. (2025). Chronic overlapping pain conditions and nociplastic pain. Hum. Genet. Genom. Adv..

[4] Malcangio M., Sideris-Lampretsas G. (2025). How microglia contribute to the induction and maintenance of neuropathic pain. Nat. Rev. Neurosci..

[5] Reuter S., Bajorat R., Müller-Graf F., Zitzmann A.R., Müller V., Pickhardt A.-L., Reuter D.A., Böhm S.H., Vollmar B. (2025). The role of microcirculatory dysfunction during paclitaxel treatment as a critical co-factor for the development of chemotherapy-induced peripheral neuropathy. Geburtshilfe Und Frauenheilkd..

[6] Carmland M.E., Kreutzfeldt M.D., Brask-Thomsen P.K., Jensen T.S., Bach F.W., Sindrup S.H., Finnerup N.B. (2025). Signs of hyperpathia in chronic peripheral neuropathic pain. Eur. J. Pain.

[7] Birangane R., Parkarwar P. (2025). Craniofacial Neuralgia.

[8] Pergolizzi J.V., LeQuang J.A.K., El-Tallawy S.N., Ahmed R.S., Wagner M., Varrassi G. (2024). The challenges in clinical diagnosis of trigeminal neuralgia: A review. Cureus.

[9] Mu G., Ren C., Zhang Y., Lu B., Feng J., Wu D., Xu X., Ou C. (2023). Amelioration of central neurodegeneration by docosahexaenoic acid in trigeminal neuralgia rats through the regulation of central neuroinflammation. Int. Immunopharmacol..

[10] Obermann M. (2019). Recent advances in understanding/managing trigeminal neuralgia. F1000Research.

[11] Amaechi O. (2025). Trigeminal neuralgia: Rapid evidence review. Am. Fam. Physician.

[12] Mayoral V., Galvez R., Ferrándiz M., Vázquez X.M., Cordero-García C., Montero A.A., Pérez C., Pérez-Páramo M. (2025). Pregabalin vs. gabapentin in the treatment of neuropathic pain: A comprehensive systematic review and meta-analysis of effectiveness and safety. Front. Pain Res..

[13] Feldman A., Weaver J. (2025). Pharmacologic and nonpharmacologic management of neuropathic pain. Semin. Neurol..

[14] Saab K., Gada U., Culakova E., Burnette B., Jorgensen C., Shah D., Morrow G., Mustian K., Sohn M.B., Edwards R.R. (2025). Personalized outcomes in neuropathic pain: A clinical relevance and assay sensitivity analysis from a randomized controlled trial. Pain Med..

[15] Pergolizzi J.V.J., LeQuang J.A., El-Tallawy S.N., Wagner M., Ahmed R.S., Varrassi G. (2024). An update on pharmacotherapy for trigeminal neuralgia. Expert Rev. Neurother..

[16] Agbo J., Ibrahim Z.G., Magaji S.Y., Mutalub Y.B., Mshelia P.P., Mhya D.H. (2023). Therapeutic efficacy of voltage-gated sodium channel inhibitors in epilepsy. Acta Epileptol..

[17] Yates J.M., Smith K.G., Robinson P.P. (2005). The effect of carbamazepine on injury-induced ectopic discharge in the lingual nerve. Brain Res..

[18] Pineda-Farias J.B., Loeza-Alcocer E., Nagarajan V., Gold M.S., Sekula R.F. (2021). Mechanisms underlying the selective therapeutic efficacy of carbamazepine for attenuation of trigeminal nerve injury pain. J. Neurosci..

[19] Xu H., Guan M., Chen Y., Qin H., Huang S. (2024). Efficacy and safety of pregabalin vs carbamazepine in patients with central post-stroke pain. Neurol. Res..

[20] Freynhagen R., Baron R., Huygen F., Perrot S. (2025). Narrative review of the efficacy and safety of the high-concentration (179mg) capsaicin patch in peripheral neuropathic pain with recommendations for clinical practice and future research. Pain Rep..

[21] Jabeen Z., Bukhari S.A., Malik S.A., Hussain G., Kamal S. (2023). Improved gut microbiota escalates muscle function rehabilitation and ameliorates oxidative stress following mechanically induced peripheral nerve injury in mice. Pak. Vet. J..

[22] Heir R., Abbasi Z., Komal P., Altimimi H.F., Franquin M., Moschou D., Chambon J., Stellwagen D. (2024). Astrocytes are the source of TNF mediating homeostatic synaptic plasticity. J. Neurosci..

[23] Chen K.-Y., Hsueh S.-C., Parekh P., Batsaikhan B., Tweedie D., Luo W., Patel C., Chiang Y.-H., Bambakidis N., Hoffer B.J. (2025). 3-Monothiopomalidomide, a new immunomodulatory imide drug (IMiD), blunts inflammation and mitigates ischemic stroke in the rat. GeroScience.

[24] Gogulescu A., Blidisel A., Soica C., Mioc A., Voicu A., Jojic A., Voicu M., Banciu C. (2024). Neurological side effects of tnf-α inhibitors revisited: A review of case reports. Medicina.

[25] Ali G., Saeed A., Khurshid A., Ahmad S., Kashtoh H., Ataya F.S., Bathiha G.E.-S., Ullah A., Khan A. (2024). Efficacy assessment of novel methanimine derivatives in chronic constriction injury-induced neuropathic model: An *in-vivo*, *ex-vivo* and *in-silico* approach. Eur. J. Pharm. Sci..

[26] Gómez C.D., Buijs R.M., Sitges M. (2014). The anti-seizure drugs vinpocetine and carbamazepine, but not valproic acid, reduce inflammatory IL-1β and TNF-α expression in rat hippocampus. J. Neurochem..

[27] Guo W., Zhang J., Feng Y. (2024). Treatment of neuropathic pain by traditional Chinese medicine: An updated review on their effect and putative mechanisms of action. Phytother. Res..

[28] Hashim M., Badruddeen U., Akhtar J., Khan M.I., Ahmad M., Islam A., Ahmad A. (2024). Diabetic neuropathy: An overview of molecular pathways and protective mechanisms of phytobioactives. Endocr. Metab. Immune Disord. Drug Targets.

[29] Faheem M., Khan A., Shah F.A. (2024). Pharmacological investigation of natural compounds for therapeutic potential in neuropathic pain. Nat. Prod. Res..

[30] Mairuae N., Noisa P., Palachai N. (2024). Phytosome-encapsulated 6-gingerol- and 6-shogaol-enriched extracts from *Zingiber officinale* roscoe protect against oxidative stress-induced neurotoxicity. Molecules.

[31] Hafeez H., Israr B., Butt M.S., Faisal M.N. (2024). Therapeutic intervention of *Opuntia Ficus Indica* (L.) fruit and seed powder against CCl 4-induced acute liver injury in Wistar rats. Pak. Vet. J..

[32] AlMasoud N., Rabail R., Alomar T.S., Munir S., Hassan S.A., Aadil R.M. (2024). Therapeutic impact of bitter gourd seed-fortified crackers on alloxan-induced diabetic rats. Pak. Vet. J..

[33] Şahin İ.O., Tunalı M.B., Aktaş A., Tüfekci K.K., Kaplan S. (2024). The effects of curcumin on hyperglycaemia-induced optic nerve damage in Wistar albino rats: An electron microscopic and stereological study. Pak. Vet. J..

[34] Zekeya N., Ibrahim M., Mamiro B., Ndossi H., Kilonzo M., Mkangara M., Chacha M., Chilongola J., Kideghesho J. (2022). Potential of natural phenolic antioxidant compounds from *Bersama Abyssinica* (Meliathacea) for treatment of chronic diseases. Saudi J. Biol. Sci..

[35] Du J., Zhao Q., Zhang Y., Wang Y., Ma M. (2012). 7,8-dihydroxycoumarin improves neurological function in a mouse model of sciatic nerve injury. Neural Regen. Res..

[36] Usman M., Malik H., Ahmed Z., Tokhi A., Arif M., Huma Z., Rauf K., Sewell R. (2023). 6,7,8-Trimethoxycoumarin attenuates vincristine induced peripheral neuropathic pain, potential role of 5HT3 and opioid receptors and monoamines. J. Xi’an Shiyou Univ. Nat. Sci. Ed..

[37] Usman M., Malik H., Tokhi A., Arif M., Huma Z., Rauf K., Sewell R.D.E. (2023). 5,7-Dimethoxycoumarin ameliorates vincristine induced neuropathic pain: Potential role of 5HT_3_ receptors and monoamines. Front. Pharmacol..

[38] Muthuraman A., Sood S., Ramesh M., Puri K.D.S., Peters A., Chauhan A., Arora P.K., Rana A. (2012). Therapeutic potential of 7,8-dimethoxycoumarin on cisplatin- and ischemia/reperfusion injury-induced acute renal failure in rats. Naunyn. Schmiedebergs Arch. Pharmacol..

[39] Lee N.K., Chung Y.C., Kang C.I., Park S., Hyun C. (2019). 7,8-dimethoxycoumarin attenuates the expression of IL-6, IL-8, and CCL2/MCP-1 in TNF-α-treated HaCaT cells by potentially targeting the NF-κB and MAPK pathways. Cosmetics.

[40] Wagner R., Myers R.R. (1996). Endoneurial injection of TNF-alpha produces neuropathic pain behaviors. Neuroreport.

[41] Sorkin L.S., Doom C.M. (2000). Epineurial application of TNF elicits an acute mechanical hyperalgesia in the awake rat. J. Peripher. Nerv. Syst..

[42] Lu Z.Y., Fan J., Yu L.H., Ma B., Cheng L.M. (2021). The up-regulation of TNF-α maintains trigeminal neuralgia by modulating MAPKs phosphorylation and BKCa channels in trigeminal nucleus caudalis. Front. Cell Neurosci..

[43] Ding W., Yang L., Chen Q., Hu K., Liu Y., Bao E., Wang C., Mao J., Shen S. (2023). Foramen lacerum impingement of trigeminal nerve root as a rodent model for trigeminal neuralgia. JCI. Insight.

[44] Damjanovska M., Cvetko E., Hadzic A., Seliskar A., Plavec T., Mis K., Vuckovic Hasanbegovic I., Stopar Pintaric T. (2015). Neurotoxicity of perineural vs intraneural-extrafascicular injection of liposomal bupivacaine in the porcine model of sciatic nerve block. Anaesthesia.

[45] Yoon C., Wook Y.T., Sik N.H., Ho K.S., Mo C.J. (1994). Behavioral signs of ongoing pain and cold allodynia in a rat model of neuropathic pain. Pain.

[46] Gupta S., Ling J., Gu J.G. (2024). Assessment of orofacial nociceptive behaviors of mice with the sheltering tube method: Oxaliplatin-induced mechanical and cold allodynia in orofacial regions. Mol. Pain.

[47] Chaplan S.R., Bach F.W., Pogrel J.W., Chung J.M., Yaksh T.L. (1994). Quantitative assessment of tactile allodynia in the rat paw. J. Neurosci. Methods.

[48] Niehaus W.G.J., Samuelsson B. (1968). Formation of malonaldehyde from phospholipid arachidonate during microsomal lipid peroxidation. Eur. J. Biochem..

[49] Beutler E., Duron O., Kelly B.M. (1963). Improved method for the determination of blood glutathione. J. Lab. Clin. Med..

[50] Muthuraman A., Ramesh M. (2016). Ischemic-reperfusion of unilateral external iliac artery in rat: A new model for vasculitic femoral neuropathy. Neurosci. Lett..

[51] Lowry O.H., Rosebrough N.J., Farr A.L., Randall R.J. (1951). Protein measurement with the Folin phenol reagent. J. Biol. Chem..

[52] Rao D., Little P., Sills R. (2013). Subsite awareness in neuropathology evaluation of national toxicology program (NTP) studies: A review of select neuroanatomical structures with their functional significance in rodents. Toxicol. Pathol..

[53] Borin A., Toledo R.N., de Faria S.D., Testa J.R.G., Cruz O.L.M. (2006). Behavioral and histologic experimental model of facial nerve regeneration in rats. Braz. J. Otorhinolaryngol..

[54] Kaplan C.M., Kelleher E., Irani A., Schrepf A., Clauw D.J., Harte S.E. (2024). Deciphering nociplastic pain: Clinical features, risk factors and potential mechanisms. Nat. Rev. Neurol..

[55] Gu D., Xia Y., Ding Z., Qian J., Gu X., Bai H., Jiang M., Yao D. (2024). Inflammation in the peripheral nervous system after injury. Biomedicines.

[56] Choudhary A.N., Tahir F. (2023). The therapeutic effect of *Gymnema Sylvestre* extract against hyperglycemia: In Vivo Study. Agrobiol. Rec..

[57] Dash U.C., Bhol N.K., Swain S.K., Samal R.R., Nayak P.K., Raina V., Panda S.K., Kerry R.G., Duttaroy A.K., Jena A.B. (2025). Oxidative stress and inflammation in the pathogenesis of neurological disorders: Mechanisms and implications. Acta Pharm. Sin. B.

[58] Kung C.-C., Dai S.-P., Yen C.-H., Lee Y.-J., Chang S.-L., Fang Y.-T., Lin H.-L., Chen C.-L. (2025). Animal neuropathic pain aroused by conglutinating oxidative regenerative cellulose on dorsal root ganglion. J. Neuropathol. Exp. Neurol..

[59] Albano E., Bellomo G., Parola M., Carini R., Dianzani M.U. (1991). Stimulation of lipid peroxidation increases the intracellular calcium content of isolated hepatocytes. Biochim. Biophys. Acta.

[60] Tumilaar S.G., Hardianto A., Dohi H., Kurnia D. (2024). A Comprehensive review of free radicals, oxidative stress, and antioxidants: Overview, clinical applications, global perspectives, future directions, and mechanisms of antioxidant activity of flavonoid compounds. J. Chem..

[61] Wongchitrat P., Chanmee T., Govitrapong P. (2024). Molecular mechanisms associated with neurodegeneration of neurotropic viral infection. Mol. Neurobiol..

[62] Kaur S., Muthuraman A. (2018). Anti-neuralgesic effect of ginsenoside rg1 (GRg) in chemotherapy-induced neuropathic pain. J. Pharm. Sci. Res..

[63] Sood S., Muthuraman A., Gill N.S., Bali M., Sharma P.D. (2010). Role of 7,8-dimethoxycoumarin in anti-secretary and anti-inflammatory action on pyloric ligation-induced gastritis in rats. J. Asian Nat. Prod. Res..

[64] Yang W., Wang H. (2018). 5,7-dimethoxycoumarin prevents chronic mild stress induced depression in rats through increase in the expression of heat shock protein-70 and inhibition of monoamine oxidase-A levels. Saudi J. Biol. Sci..

[65] Cheriyan B.V., Shanmugasundaram J., Ramakrishnan P., Ramasamy K., Karthikeyan R., Venkataraman S., Roy A., Parthasarathy P.R. (2024). Exploring the potential therapeutic benefits of 7-methoxy coumarin for neuropathy pain: An in vivo, in vitro, and in silico approach. Mol. Biol. Rep..

[66] Li Z., Hu J., Sun M., Song X., Li G., Liu Y., Li G., Ji H., Liu G., Chen N. (2013). In vitro and in vivo anti-inflammatory effects of IMMLG5521, a coumarin derivative. Int. Immunopharmacol..

[67] Kim K.N., Yang H.W., Ko S.C., Ko Y.J., Kim E.A., Roh S.W., Ko E.Y., Ahn G., Heo S.J., Jeon Y.J. (2014). 6-7-Dimethoxy-4-methylcoumarin suppresses pro-inflammatory mediator expression through inactivation of the NF-κB and MAPK pathways in LPS-induced RAW 264.7 cells. EXCLI J..

